# Functional outcomes of surgical treatment of ureteral injury following gynecological and obstetrical surgery

**DOI:** 10.1016/j.amsu.2022.104067

**Published:** 2022-06-28

**Authors:** Moez Rahoui, Yassine Ouanes, Kays Chaker, Kheireddine Mourad Dali, Mokhtar Bibi, Ahmed Sellami, Sami Ben Rhouma, Yassine Nouira

**Affiliations:** Urology Department La Rabta Hospital, Tunis, Tunisia

**Keywords:** Iatrogenic ureteral injury, Ureteral ligation, Hysterectomy, Ureterovesical reimplantation

## Abstract

**Introduction:**

Iatrogenic ureteral lesions represent one of the serious complications that can follow obstetric and gynecological surgery. This condition has a fatal consequence on renal function if it's not promptly diagnosed and managed.

**Objective:**

The aim of our study was to report our experience in the management of this pathology.

****Materials & Methods**:**

This is a retrospective study of 32 patients treated for an iatrogenic ureteral injury after gynecological or obstetrical surgery, collected in the urology department of the Rabta Hospital over a 15-year period (2005–2020). Clinical presentation, investigations, and operative and postoperative details were reviewed from the patients' charts.

**Results:**

The average age of the patients was 42.6 (21–61). Multiparity was observed in 90.6% of cases. Hysterectomy was the most common cause (71.87%), followed by cesarean operation (18.75%), mainly for patients with placenta percreta (12.5%), and lastly, cure of prolapse by the upper approach in 9.37% of cases. The symptoms were dominated by low back pain and urinary incontinence. Stenosis was the most frequent lesion in 25 cases, followed by a section in 4 cases. A ureterovaginal fistula was observed in 3 case s. The first-line treatment of the patients was drainage by a ureteral stent (15.6%) or by a percutaneous nephrostomy (84.4%). Ureterovesical reimplantation was performed in 26 cases (81.25%). However, one patient had an Ileal ureter replacement. During follow-up, treatment failure was noted in 7 patients. Four patients developed secondary hydronephrosis treated with a urethral stent while 3 patients required nephrectomy. The type of gynecological and obstetrical procedure (open hysterectomy), history of pelvic surgery, and malignant pathology were predictive factors of treatment failure.

**Conclusions:**

Injuries to the ureter during gynecological and obstetrical surgery are generally rare. The diversity of repair techniques and the contribution of endo-urological techniques most often allow renal preservation, knowing that the best treatment remains prevention.

## Highlights

• Iatrogenic ureteral lesions represent one of the serious complications that can follow obstetric and gynecological surgery.
• This condition has a fatal consequence on renal function if it's not promptly diagnosed and managed.
• The diversity of repair techniques and the contribution of endo-urological techniques most often allow renal preservation.

## 1 Introduction

The ureter is a retroperitoneal organ running along with the psoas muscle in contact with the posterior parietal peritoneum [1]. It presents intimate relations with the female genital tract in its pelvic portion [1]. This anatomical specificity exposes the ureter to several traumatic injuries during pelvic surgery in women.

For several years, gynecological and obstetrical surgery has been the most frequent source of ureteral injuries, representing almost 75% of iatrogenic trauma to the ureter [2]. However, the incidence of ureteral injury remains low, in the order of 0.5–1% of pelvic surgeries and about 0.5% of hysterectomies [2].

These injuries observed in gynecological and obstetric procedures occur in both open and laparoscopic surgery, although the management of the injuries differs [3]. These injuries can have serious consequences if not diagnosed and treated immediately. They can be life-threatening and may affect the functional prognosis of the kidney. The first treatment is essentially based on endoscopy but in case of failure, open surgery represents a suitable but more invasive solution [2,4].

Several authors have been interested in studying ureteral lesions observed in gynecological and obstetrical surgery. The conclusions diverge as to the diagnostic strategy, the therapeutic management, and the functional outcomes after the treatment.

This inspired us to conduct this study, which aims to report our experience in the management of ureteral trauma following gynecological and obstetric surgery. Secondly, we determined the functional outcomes of surgical treatment of this rare condition.

## 2 Patients and methods

It was a retrospective, observational study conducted in a tertiary care center. Institutional Review Board approval was obtained (CEBM.EPS.HR/62/2020). Our data has been reported in line with the STROCSS criteria [5]. In this study, the authors confirmed that all methods were carried out under the relevant guidelines and regulations (Helsinki Declaration) under the number researchregistry 7911.

We retrospectively included all patients who were referred to our department for management of a ureteral injury following gynecological or obstetric surgery, during a 15-year period from January 2005 to December 2020. We excluded patients who underwent surgery in other departments and were transferred to us for postoperative monitoring.

A systematic review of patient records and operative reports was performed. The data analyzed were: age, medical and surgical history, type of surgery, circumstances of discovery, time of diagnosis, results of biological and radiological examinations, type of urological surgery, duration of hospitalization, complications, and radiological and biological examinations during monitoring.

We defined the success of the treatment as the absence of recurrence (scarring) and secondary hydronephrosis on the three-month postoperative CT scan. The need for reintervention or nephrectomy was considered a treatment failure. Patients were divided into two groups according to the success or failure of the treatment.

Statistical analysis was done using the SPSS v.22 programs. Differences between the groups were analyzed using the Wilcoxon rank-sum test for continuous variables and chi-square or Fisher's exact test for categorical variables, with P < 0.05 considered to indicate statistical significance.

## 3 Results

A total of 41 patients treated for ureteral injury secondary to gynecological and obstetric surgery during the study period were initially included. After the application of the non-inclusion criteria, 9 patients were excluded. Finally, the 32 patients who met the inclusion criteria were selected for this study.

The mean age of the patients was 44.4 years, with extremes of 24 and 61 years. The average time to diagnosis was 4.23 days with extremes ranging from 1 to 14 days. Multiparity was found in 90.6% of cases. A history of pelvic surgery was noted in 8 patients. The circumstances of discovery were back pain in 23 cases, renal failure in 3 cases, urinary fistula in 3 cases, fever in 2 cases, and hematuria in one case. Hysterectomy was the most common cause (71.87%), followed by cesarean operation (18.75%), mainly for patients with placenta percreta (12.5%), and lastly, cure of prolapse by the upper approach in 9.37% of cases. Concerning hysterectomy, 14 were by open approach and 9 by laparoscopic approach. Malignant pathology was found in 8 patients. Ultrasound showed dilatation of the upper urinary tract in 25 cases. CT scans and intravenous urography confirmed the diagnosis in all cases. Stenosis was the most frequent lesion in 25 cases, followed by a section in 4 cases. A ureterovaginal fistula was observed in 3 cases. The first-line treatment for the patients was drainage by a ureteral stent (15.62%) or by a percutaneous nephrostomy (84.38%). Ureterovesical reimplantation was performed in 26 cases (81.25%). However, one patient had an Ileal ureter replacement. The average duration of hospitalization was 7.2 days with extremes ranging from 4 to 11 days. The baseline characteristics of the patients are summarized in Table 1.

**Table 1:** Epidemiological and clinical characteristics of patients.

Variables	Value
Age, mean	44.4 [24–61]
Diabetes mellitus, n, %	
**Yes**	9 (28.1)
**No**	23 (71.9)
Hypertension, n, %	
**Yes**	7 (21.9)
**No**	25 (78.1)
Multiparity, n, %	
**Yes**	29 (90.6)
**No**	3 (9.4)
History of pelvic surgery, n, %	
**Yes**	8 (25)
**No**	24 (75)
The circumstances of discovery, n, %	
**Back pain**	23 (71.9)
**Renal failure**	3 (9.4)
**Urinary fistula**	3 (9.4)
**Fever**	2 (6.3)
**Hematuria**	1 (3.2)
Gynecological and obstetrical procedure, n, %	
**Open hysterectomy**	14 (4.4)
**Laparoscopic hysterectomy**	9 (28.1)
**Cesarean operation**	6 (18.8)
**Cure of prolapse**	3 (9.4)
Type of lesion, n, %	
**Stenosis**	25 (78.1)
**Section**	4 (12.5)
**Urinary fistula**	3 (9.4)
First-line treatment, n, %	
**Ureteral stent**	5 (15.6)
**Percutaneous Nephrostomy**	27 (84.4)
Definitive treatment, n, %	
**Ureterovesical reimplantation**	26 (81.1)
**Ileal ureter replacement**	1 (3.1)

The average follow-up time was 36.2 months with extremes ranging from 24 to 126 months. During follow-up, treatment failure was noted in 7 patients (21.87%). Four patients developed secondary hydronephrosis treated with a urethral stent while 3 patients required nephrectomy. In addition, chronic renal failure was noted in two patients. Statistical analysis showed no significant differences between the two groups in terms of age, time to diagnosis, BMI, diabetes, hypertension, type of initial treatment, and definitive treatment. However, there were significant differences between the two arms in terms of the type of gynecological and obstetrical procedure (open hysterectomy), history of pelvic surgery, and malignant pathology (Table 2).

**Table 2:** Comparison of patients according to the outcomes of surgical treatment.

Variables	Treatment success	Treatment failure	P-value
Number of patients	25(78.1)	7 (21.9)	N/A
Age, mean	43.9	44.1	0.78
Diabetes mellitus, n, %	7 (28)	2 (28.5)	0.84
Hypertension, n, %	6 (24)	1 (14.3)	0.21
History of pelvic surgery, n, %	3(12)	5 (71.4)	0.04
Multiparity, n, %	21 (84)	4 (57.1)	0.07
malignant pathology, n, %	4(16)	4(57.1)	0.03
Gynecological and obstetrical procedure, n, %			
**Open hysterectomy**	8 (32)	'	0.001
**Laparoscopic hysterectomy**	7 (28)	2 (28.5)	0.89
**Cesarean operation**	5 (20)	1 (14.3)	0.23
**Cure of prolapse**	3 (12)	0 (0)	>0.99
Type of lesion, n, %			
**Stenosis**	20 (80)	5 (71.4)	0.72
**Section**	3 (12)	1 (14.3)	
**Urinary fistula**	2 (8)	1 (14.3)	
First-line treatment, n, %			
**Ureteral stent**	5 (20)	0 (0)	>0.99
**Percutaneous Nephrostomy**	20 (80)	7 (100)	
Definitive treatment, n, %			
**Ureterovesical reimplantation**	19 (76)	7 (100)	>0.99
**Ileal ureter replacement**	1 (4)	0 ()	

## 4 Discussion

The overall estimate of the frequency of operative lesions of the ureter is very variable according to the series analyzed, ranging from 0.5 to 30% [2,3]. In fact, this frequency depends on several factors. The degree of medicalization plays a very important role and iatrogenic urinary complications of obstetrical origin are mainly the prerogative of developing countries [4]. The type of surgery performed: whether abdominal or vaginal surgery is performed, the ureter is always at risk [6]. Symmonds found 2–3% of ureteral lesions after hysterectomy, while Robert reports 3.6% [4]. Enlarged hysterectomies with lymphadenectomy (Wertheim type) are the biggest providers of ureteral lesions: 10–30% of cases [4]. However, laparoscopic surgery does not spare this organ; Chapron reports a rate of 1.7% of ureteral injury during laparoscopic surgery [7]. In our series, Hysterectomy was the most common cause (71.87%), followed by cesarean operation (18.75%), mainly for patients with placenta percreta (12.5%), and lastly, cure of prolapse by the upper approach in 9.37% of cases.

In developing countries, it is mainly young and primiparous women who are interested in gynaeco-obstetric ureteral injury, whereas in developed countries these lesions are the prerogative of multiparous women [2.4]. In our study, multiparity was found in 90.6% of cases. The early diagnosis conditions the therapeutic result [1,2,6]. Ideally, this accident should be recognized during the operation but this is rare. According to Cussenot et al. [8], the diagnosis was made intraoperatively in 20% of cases while Benoit et al. [9] reported that intraoperative diagnosis was made in 10% of cases. In our series, the average time to diagnosis was 4.23 days with extremes ranging from 1 to 14 days.

The presenting signs are mainly urine leakage and back pain, rarely anuria or a pelvic mass (indicating a urinoma) or hematuria. In different series [[1], [2], [3],^6^], urinary leakage through the vagina is the most frequent revealing sign, followed by back pain. In our study, Back pain was the most common sign followed by urinary leakage. If the revealing signs are generally very noisy, the clinical examination is poor apart from the observation of urine flow through the vagina, through the drainage system or through the surgical wound. Palpation looks for pelvic impaction, which indicates urine effusion. Vaginal examination reveals, in case of urine leakage, the external orifice of the fistula and looks for a possible associated vesicovaginal fistula. The methylene blue test confirms the integrity of the bladder unless there is an associated vesicovaginal fistula [2,4]. Additional examinations will help to clarify the diagnosis and to assess the function and integrity of the contralateral urinary tract. Ultrasound, a non-invasive examination, has become the first line of investigation: it shows dilatation of the excretory tract and gives an idea of the parenchymal index [4,10]. In our research, ultrasound showed dilatation of the upper urinary tract in 25 cases.

Intravenous Urography was the essential examination for the positive diagnosis of ureteral injuries [4,10]. It allows for determining the impact of the ureteral trauma on the upper urinary tract, assessing the condition of the contralateral kidney, and determining the site of the ureteral trauma. Nephrostomy with opacification allows the ureter to be visualized if retrograde catheterization is not possible. Currently, a Ct scan is the examination of choice for demonstrating urinary leakage and locating its location [1,2]. In our study, the combination of CT scan and intravenous urography allowed us to confirm the diagnosis and establish the lesion in all cases. Nuclear magnetic resonance imaging (MRI) has the advantage of being non-irradiating and having a better contrast resolution. The examination includes T1, T2, and T1-weighted sequences after injection of gadolinium [4,11]. The sensitivity of MRI in the diagnosis of iatrogenic ureteral lesions is around 86.8% [4,12]. The search for a urinary infection and the assessment of renal function should be systematic in these situations. In our series, renal failure was noted in 2 cases.

The aim of treatment is to restore ureteral continuity. This will depend on several parameters. Firstly, the anatomical data are important, especially the state of the ureter (especially the distal part), the state of the kidney, the bladder, and associated lesions [2,4]. Secondly, the condition of the patient, i.e. age, general state, type of initial disease which led to the operation responsible for the lesion, and finally the technical means available to the surgeon and his competence [4]. Endoscopic methods often allow a simple approach to the pathology and have a diagnostic and therapeutic role. Retrograde ureteral catheterization is the first initiative. If this fails, the percutaneous approach with anterograde catheterization of the excretory tract is used. Open surgery is indicated if endourological treatment fails [2,6,8]. Surgical treatment consists of either a simple repair of the damaged organ or ureterovesical reimplantation possibly associated with more or less complex devices [2,13,14]. In our series, 5 patients had ureteral stent drainage. The success rate was 100%. In the remaining cases, after initial drainage by percutaneous nephrostomy, 26 patients had ureterovesical reimplantation and one patient had Ileal ureter replacement.

The prognosis depends on the anatomical conditions (state of the ureter and associated lesions), the time taken for treatment, the general condition of the woman, and the experience of the surgeon [2,4]. In our series, treatment failure was noted in 7 patients (21.87%). Four patients developed secondary hydronephrosis treated with a urethral stent while 3 patients required nephrectomy. Open hysterectomy, previous pelvic surgery, and malignancy have been identified as predictors of treatment failure. In addition, chronic renal failure was noted in two patients.

Before interpreting the results, our study has several limitations that need to be recognized. Firstly, this study is monocentric and based on a limited population. Second, the retrospective design was not ideal for achieving the study objectives. Despite these limitations, this study demonstrated that open hysterectomy, previous malignant surgery, and neoplastic pathology were predictive of failed treatment of ureteral lesions secondary to gynecological and obstetric surgery. Finally, a large-scale, multicenter, prospective study is needed to confirm these results.

## 5 Conclusions

The consequences of ureteral injury are serious and any surgeon approaching the pelvis should be aware of this risk as the urinary and genital systems are intimately linked from the embryonic stage. Awareness of this risk should prompt greater caution, especially when the anatomical relationships of the pelvis are altered by inflammatory processes, tumors, or endometriosis. The practice of preoperative catheterization in case of high-risk surgery remains the best wisdom. Early diagnosis based on regular postoperative monitoring allows adequate management to preserve renal function.

## Provenance and peer review

Not commissioned, externally peer reviewed.

## Sources of funding

This research did not receive any specific grant from funding agencies in the public, commercial, or not-for-profit sectors.

## Ethical approval

Not applicable.

## Consent

Written informed consent was obtained from the patient for publication of this case report and accompanying images. A copy of the written consent is available for review by the Editor-in-Chief of this journal on request.

## Author contribution

Rahoui Moez, Yassine ouannes and Kays chaker: Data collection, Manuscript writing, Results discussion.

Kheireddine Mourad daly, Bibi Mokhtar and Ahmed sellami: Manuscript writing and revision.

Ben rhouma sami and Nouira yassine: Paper revision.

## Registration of research studies

1. Name of the registry:: N/a
2. Unique identifying number or registration ID:: N/a
3. Hyperlink to your specific registration (must be publicly accessible and will be checked):: N/a

## Guarantor

Rahoui Moez is the guarantor of the study and accept full responsibility for the work and/or the conduct of the study, had access to the data and controlled the decision to publish.

## Declaration of competing interest

Authors do not report any conflict of interest.
